# Supplementary material for: Does training method matter? Evidence for the negative impact of aversive-based methods on companion dog welfare
Source: PLoS One. 2020 Dec 16;15(12):e0225023. doi: 10.1371/journal.pone.0225023 (PMC7743949; doi:10.1371/journal.pone.0225023)
Supplement: S1 Appendix — For each training session, the number of intended positive punishments and negative reinforcements was divided by the total number of intended positive punishments, negative reinforcements, positive reinforcements and negative punishments. Schools A and D were categorized as Group Aversive, Schools C and F as Group Mixed and Schools B, E and G as Group Reward. (DOCX) [file pone.0225023.s001.docx]

Appendix S1. Proportion (mean ± standard deviation) of intended aversive-based techniques used during the six training sessions analyzed for each training school. For each training session, the number of intended positive punishments and negative reinforcements was divided by the total number of intended positive punishments, negative reinforcements, positive reinforcements and negative punishments. Schools A and D were categorized as Group Aversive, Schools C and F as Group Mixed and Schools B, E and G as Group Reward.

|  | **Dog training school** | | | | | | |
| --- | --- | --- | --- | --- | --- | --- | --- |
| **Session** | **A** | **B** | **C** | **D** | **E** | **F** | **G** |
| **1** | 0.75 | 0.00 | 0.34 | 0.90 | 0.00 | 0.37 | 0.00 |
| **2** | 0.87 | 0.00 | 0.24 | 0.76 | 0.00 | 0.18 | 0.00 |
| **3** | 0.62 | 0.00 | 0.09 | 0.76 | 0.00 | 0.51 | 0.00 |
| **4** | 0.61 | 0.00 | 0.10 | 0.74 | 0.00 | 0.11 | 0.00 |
| **5** | 0.81 | 0.00 | 0.19 | 0.87 | 0.00 | 0.58 | 0.00 |
| **6** | 0.92 | 0.00 | 0.36 | 0.98 | 0.00 | 0.45 | 0.00 |
| **Mean** | 0.76 | 0.00 | 0.22 | 0.84 | 0.00 | 0.37 | 0.00 |
| **Standard deviation** | 0.13 | 0.00 | 0.12 | 0.10 | 0.00 | 0.19 | 0.00 |
