# Supplementary material for: Does training method matter? Evidence for the negative impact of aversive-based methods on companion dog welfare
Source: PLoS One. 2020 Dec 16;15(12):e0225023. doi: 10.1371/journal.pone.0225023 (PMC7743949; doi:10.1371/journal.pone.0225023)
Supplement: S3 Appendix — S3a Table. Analysis of Generalized Estimating Equation for the stress-related behaviors analysis. S3b Table. Analysis of Generalized Estimating Equation for the behavioral state analysis. S3c Table. Analysis of Generalized Estimating Equation for the panting analysis. S3d Table. Solutions for fixed effects from the generalized linear mixed model for the cognitive bias analysis. (DOCX) [file pone.0225023.s003.docx]

Appendix S3. Negative binomial generalized linear model details.

Table S3a. Analysis of Generalized Estimating Equation for the stress-related behaviors analysis.

| **Dependent Variable** | **Effect** | **Estimate** | **Standard Error** | **Z** | **Pr > \|Z\|** |
| --- | --- | --- | --- | --- | --- |
| Body Turn | Intercept | -0.3923 | 0.2654 | -1.48 | 0.1394 |
|  | Group Aversive | 2.1908 | 0.2963 | 7.39 | <0.0001 |
|  | Group Mixed | 1.6889 | 0.2622 | 6.44 | <0.0001 |
|  | Group Reward | 0.0000 | 0.0000 | n.a. | n.a. |
|  | Dog Age | -0.0252 | 0.0099 | -2.55 | 0.0107 |
|  | Children Not Present | -0.5888 | 0.2060 | -2.37 | 0.0178 |
|  | Children Present | 0.0000 | 0.0000 | n.a. | n.a. |
| Move Away | Intercept | 0.9859 | 0.1395 | 7.07 | <0.0001 |
|  | Group Aversive | 0.4617 | 0.2034 | 2.27 | 0.0232 |
|  | Group Mixed | 0.3806 | 0.2382 | 1.60 | 0.1101 |
|  | Group Reward | 0.0000 | 0.0000 | n.a. | n.a. |
| Crouch | Intercept | -0.5274 | 0.2783 | -1.89 | 0.0581 |
|  | Group Aversive | 1.7963 | 0.3776 | 4.76 | <0.0001 |
|  | Group Mixed | 1.1306 | 0.3667 | 3.08 | 0.0020 |
|  | Group Reward | 0.0000 | 0.0000 | n.a. | n.a. |
| Yawn | Intercept | -0.9814 | 0.3463 | -2.83 | 0.0046 |
|  | Group Aversive | 2.3215 | 0.3535 | 6.57 | <0.0001 |
|  | Group Mixed | 1.0856 | 0.4538 | 2.39 | 0.0167 |
|  | Group Reward | 0.0000 | 0.0000 | n.a. | n.a. |
|  | Dog Age | -0.0348 | 0.0126 | -2.77 | 0.0056 |
| Lip Lick | Intercept | 1.4126 | 0.1103 | 12.81 | <0.0001 |
|  | Group Aversive | 2.6110 | 0.1569 | 16.64 | <0.0001 |
|  | Group Mixed | 1.4691 | 0.2004 | 7.33 | <0.0001 |
|  | Group Reward | 0.0000 | 0.0000 | n.a. | n.a. |
| Body Shake | Intercept | -0.8817 | 0.2565 | -3.44 | 0.0006 |
|  | Group Aversive | 0.7425 | 0.2617 | 2.84 | 0.0046 |
|  | Group Mixed | 0.0205 | 0.3351 | 0.06 | 0.9512 |
|  | Group Reward | 0.0000 | 0.0000 | n.a. | n.a. |
|  | Children Not Present | 0.5774 | 0.2386 | 2.42 | 0.0155 |
|  | Children Present | 0.0000 | 0.0000 | n.a. | n.a. |

| **Score statistics for type III Generalized Estimating Equation Analysis** | | | | |
| --- | --- | --- | --- | --- |
| **Dependent Variable** | **Effect** | **Num DF** | **Chi-Square** | **Pr > Chi-Square** |
| Body Turn | Group | 2 | 25.89 | <0.0001 |
|  | Dog Age | 1 | 4.47 | 0.0344 |
|  | Children | 1 | 3.83 | 0.0504 |
| Move Away | Group | 2 | 5.25 | 0.0725 |
| Crouch | Group | 2 | 13.26 | 0.0013 |
| Yawn | Group | 2 | 19.64 | <0.0001 |
|  | Dog Age | 1 | 4.35 | 0.037 |
| Lip Lick | Group | 2 | 35.87 | <0.0001 |
| Body Shake | Group | 2 | 5.82 | 0.0545 |
|  | Children | 1 | 5.30 | 0.0214 |

n.a.=not applicable.

Table S3b. Analysis of Generalized Estimating Equation for the behavioral state analysis.

| **Dependent Variable** | **Effect** | **Estimate** | **Standard Error** | **Z** | **Pr > \|Z\|** |
| --- | --- | --- | --- | --- | --- |
| Excited | Intercept | 2.2192 | 0.0602 | 36.84 | <0.0001 |
|  | Group Aversive | -1.8875 | 0.2104 | -8.97 | <0.0001 |
|  | Group Mixed | -0.4679 | 0.1110 | -4.21 | <0.0001 |
|  | Group Reward | 0.0000 | 0.0000 | n.a. | n.a. |
|  | Training Session 1 | 0.2404 | 0.0732 | 3.28 | 0.0010 |
|  | Training Session 2 | 0.0433 | 0.0726 | 0.60 | 0.5506 |
|  | Training Session 3 | 0.0000 | 0.0000 | n.a. | n.a. |
| Relaxed | Intercept | 0.9923 | 0.2219 | 4.47 | <0.0001 |
|  | Group Aversive | -1.1194 | 0.4047 | -2.77 | 0.0057 |
|  | Group Mixed | -0.0254 | 0.2307 | -0.11 | 0.9124 |
|  | Group Reward | 0.0000 | 0.0000 | n.a. | n.a. |
|  | Training Session 1 | -0.4816 | 0.3220 | -1.50 | 0.1347 |
|  | Training Session 2 | 0.0586 | 0.2795 | 0.21 | 0.8339 |
|  | Training Session 3 | 0.0000 | 0.0000 | n.a. | n.a. |
| Tense | Intercept | -0.4865 | 0.1709 | -2.85 | 0.0044 |
|  | Group Aversive | 2.5925 | 0.1777 | 14.59 | <0.0001 |
|  | Group Mixed | 1.6936 | 0.2231 | 7.59 | <0.0001 |
|  | Group Reward | 0.0000 | 0.0000 | n.a. | n.a. |
| Low | Intercept | -3.0740 | 0.8063 | -3.81 | 0.0001 |
|  | Group Aversive | 2.9413 | 0.7610 | 3.86 | 0.0001 |
|  | Group Mixed | 1.1397 | 0.8062 | 1.41 | 0.1575 |
|  | Group Reward | 0.0000 | 0.0000 | n.a. | n.a. |
|  | Children Not Present | -1.1801 | 0.4610 | -2.56 | 0.0105 |
|  | Children Present | 0.0000 | 0.0000 | n.a. | n.a. |

| **Score statistics for type III Generalized Estimating Equation Analysis** | | | | |
| --- | --- | --- | --- | --- |
| **Dependent Variable** | **Effect** | **Num DF** | **Chi-Square** | **Pr > Chi-Square** |
| Excited | Group | 2 | 50.34 | <0.0001 |
|  | Training Session | 2 | 10.33 | 0.0057 |
| Relaxed | Group | 2 | 12.26 | 0.0022 |
|  | Training Session | 2 | 5.13 | 0.0769 |
| Tense | Group | 2 | 52.77 | <0.0001 |
| Low | Group | 2 | 9.87 | 0.0072 |
|  | Children | 1 | 4.32 | 0.0377 |

n.a.=not applicable.

Table S3c. Analysis of Generalized Estimating Equation for the panting analysis.

| **Dependent Variable** | **Effect** | **Estimate** | **Standard Error** | **Z** | **Pr > \|Z\|** |
| --- | --- | --- | --- | --- | --- |
| Panting | Intercept | 0.9228 | 0.1589 | 5.81 | <0.0001 |
|  | Group Aversive | 0.9062 | 0.1954 | 4.64 | <0.0001 |
|  | Group Mixed | 0.5566 | 0.2266 | 2.46 | 0.0140 |
|  | Group Reward | 0.0000 | 0.0000 | n.a. | n.a. |

| **Score statistics for type III Generalized Estimating Equation Analysis** | | | | |
| --- | --- | --- | --- | --- |
| **Dependent Variable** | **Effect** | **Num DF** | **Chi-Square** | **Pr > Chi-Square** |
| Panting | Group | 2 | 17.08 | 0.0002 |

n.a.=not applicable.

Table S3d. Solutions for fixed effects from the generalized mixed model for the cognitive bias analysis.

| **Dependent Variable** | **Effect** | **Estimate** | **Standard Error** | **t** | **Pr > \|t\|** |
| --- | --- | --- | --- | --- | --- |
| Latency to reach bowl | Intercept | 0.9654 | 0.0694 | 13.91 | <0.0001 |
|  | Group Aversive | 0.2174 | 0.0845 | 2.57 | 0.0106 |
|  | Group Mixed | 0.1811 | 0.0925 | 1.96 | 0.0511 |
|  | Group Reward | 0.0000 | n.a. | n.a. | n.a. |
|  | Bowl Location M | 0.3388 | 0.0692 | 4.90 | <0.0001 |
|  | Bowl Location NN | 0.6478 | 0.0692 | 9.37 | <0.0001 |
|  | Bowl Location NP | -0.0064 | 0.0692 | -0.09 | 0.9261 |
|  | Bowl Location N | 1.4428 | 0.0692 | 20.86 | <0.0001 |
|  | Bowl Location P | 0.0000 | n.a. | n.a. | n.a. |

| **Type III Tests of Fixed Effects** | | | | |
| --- | --- | --- | --- | --- |
| **Dependent Variable** | **Effect** | **Num DF** | **F** | **Pr > F** |
| Latency to reach bowl | Group | 2 | 3.89 | 0.0214 |
|  | Bowl Location | 4 | 150.69 | <0.0001 |

n.a.=not applicable.
