# Supplementary material for: Does training method matter? Evidence for the negative impact of aversive-based methods on companion dog welfare
Source: PLoS One. 2020 Dec 16;15(12):e0225023. doi: 10.1371/journal.pone.0225023 (PMC7743949; doi:10.1371/journal.pone.0225023)
Supplement: Attachment — Submitted filename: Response to reviewers.docx [file pone.0225023.s004.docx]

**RESPONSE TO REVIEWERS**

We appreciate all the constructive criticism provided by the editor and the two anonymous reviewers. We have reconsidered the statistical analysis of our data as outlined in more detail in the answer to the specific comments below. Briefly, this resulted in a significant revision which lead us to include Dr. Gabriela M Morello as co-author. Following your considerations, we also decided to alter data analysis and presentation to include three training groups. Specifically, in the current version of the manuscript, we sub-divided the aversive-based schools in two groups according to the relative proportion of aversive‑based methodologies used: Group Aversive, which includes the two schools using the highest levels of aversive stimuli and Group Mixed, which includes the two schools using the lowest proportions of aversive stimuli. We believe that this alteration allows for a more detailed interpretation of the actual effects of different training methods on dog welfare and significantly improves our paper.

In what follows, we present detailed responses to all the comments.

Ethics approval information

First, we wish to clarify the situation regarding ethical approval for the study. The study was designed to measure the behavior of the dogs and not that of the owners or trainers. Accordingly, the request for approval was sent to the institutional animal ethics and welfare body, which would normally raise the question of additional ethics approval if they perceive this to be relevant, but did not do so in this case. The fact that human behavior data is not included in the ethics approval for this study also means that it is not possible for us to collect data on aversive stimuli used for each of the research subjects during each of the training session. Taking into account your relevant point that the study implies partial disclosure as regards the training schools, we are now debriefing the schools. It is of course very important to avoid that any individual school is identifiable in the paper, and for that reason we have removed details in Appendix S1 which may be unique for a school (such as when training takes place) but is not relevant data for the research paper. Our study included seven schools from a region in which there are about twenty dog training schools, and after we have edited out the details in S1 it is not possible to identify any individual school from the data we have provided.

Editor Comments

1. Statistical Methods
   1.1. *I would ask you to consider whether you could use more sophisticated statistics that would allow you to examine possible interactions*

In the current version of the manuscript, all the behavioral data (stress-related behaviors, overall behavioral state and panting) was analyzed with negative binomial regressions, with Group (Aversive, Mixed, Reward), training session (Session 1, Session 2, Session 3) and Group*Training session as factors.

1.2. *As well, as the reviewer points out, the sheer number of statistical tests run greatly inflates your risk of committing Type II errors. While I noted that you mention using Bonferonni corrections in Table S3b, there appears to be no use of a correction for multiple comparisons elsewhere- e.g., when reporting your significant correlations.*

In order to clarify where Bonferonni corrections were used we added in the Statistical analyses section “When multiple comparisons were performed, α was set at α=0.05/number of comparisons (Bonferroni correction). Specifically, post-hoc pairwise comparisons were performed for the post‑training variation in cortisol concentration, the number of trials to criterion and the adjusted latencies to the ‘middle’ location in the cognitive bias task with α=0.017. Post-hoc pairwise comparisons were also performed for the effects of dog-related variables in the dependent variables measured in the present study (see Appendix S3)” (lines 420-425).

2. Training categories
   2.1. *you do not differentiate between the schools on the basis of relative amount of positive reinforcement used by these “aversive” schools.*

The current version of the manuscript includes this information. Namely, we analyzed the number of “intended” positive reinforcers and negative punishers in addition to the number of “intended” positive punishers and negative reinforcers, and then calculated the proportion of “intended” aversive-based techniques: (number of intended positive punishments + number of intended negative reinforcements)/total number of intended operant conditioning procedures. We recognized that this analysis was more informative for presenting and interpreting the differences between the different aversive-based schools.

2.2. *As well, when such techniques are used (timing during learning) could affect stress in the dogs.*

We entirely agree with editor in this comment and this was the reason why we controlled for this factor in our study. In our inclusion criteria, all dogs had “to have attended the training school for less than two months, in order to mitigate habituation to training methods”. With this, we intended to ensure that all dogs were in an initial (and similar) phase of learning.

2.3. *While you address some of this in your Discussion, I would like you to consider how you might be able to integrate this more fully into your results and interpretation of the results.*

We agree with both the Editor and Reviewer 1 in that grouping schools that use highly different proportions of aversive-based techniques during training was an oversimplification of the issue under study. Hence, in the current version of the manuscript, the original Group Aversive was further divided in two groups: Group Aversive (composed by schools A and D, which had the two highest proportions of intended aversive-based techniques: 0.76 and 0.84, respectively), and Group Mixed (composed by schools C and F, which had the two intermediate proportions of intended aversive-based techniques: 0.22 and 0.37, respectively).

2.4. *Related to the above, please provide some more clarity around the 4 videotaped sessions you used to categorize the schools’ training methods. Did this occur prior to the beginning of dog/owner recruitment/video-recording? In your results, when you correlate the number of aversive stimuli used by schools with behaviour, cortisol, etc., are you using the data collected during these sessions? If so, have you considered using data on the number of aversives used during training for your actual subjects? Why or why not? I could argue that you would make a much stronger case if you had measures of the numbers of aversives used per dog in each school to correlate with the dog’s behaviours/cortisol. Please comment on this.*

We are now aware that our original description was not sufficiently clear. The videotaped sessions used to categorize the training methods were randomly selected among all videotaped sessions and were thus used as actual data for the present study. We hope to have made it sufficiently clear in the current version of the manuscript. In lines 118-121, one can now read “We performed an objective assessment of the training methods used by each school. To this end, we randomly selected six video recordings of training sessions per training school (see below, section 2.4.1) and analyzed the videos for the frequency of the intended operant conditioning procedures utilized during training”. In order to make our case stronger we decided to analyze two more sessions per training school; hence the six instead of four training sessions. However, the addition of two extra training sessions did not alter the degree of aversive stimuli used in each school found in the original version of the paper.

In the present version of the manuscript, as we opted for dividing the dogs in three rather than two groups, we no longer have correlation analyzes between the frequency of aversive stimuli and the different welfare indicators; the new division already takes into account the different degrees of the use of aversive stimuli. Yet, we would like to clarify that in the previous version of the paper we did correlate the different welfare measures with the number of aversive stimuli used by the training schools during the 4 (at the time) randomly picked training sessions. Although we agree that a much stronger case would be made if we had the number of aversive stimuli used per dog in each school, there were ethical reasons behind the decision of not doing so. The study was designed to measure the behavior of the dogs and not that of the owners or trainers. Accordingly, we only obtained ethical approval from the institutional animal ethics and welfare body. The fact that human behavior data is not included in the ethics approval for this study means that it is not possible for us to collect data on aversive stimuli on the level of individual owner-dog pairs.

3. Cognitive Bias Test
   3.1. *Reviewer 1 suggests that you have possibly over-interpreted the CB test results and I tend to agree. This test can be influenced by both acute and long-term stressors. As suggested, please add some information to your Discussion around interpretation issues of the test itself. Even if you effectively argue for interpreting the CB test results as indicators of long-term welfare, this is unlikely due to welfare based solely on training methods used earlier. This is an over-simplification since you clearly show that your two groups differ in other characteristics that might be expected to influence cognitive bias- e.g., as in Appendix S3, bred, age, age when separated from mother, owner/main trainer gender, and whether more than one dog lives in the home. These factors may impact measures of welfare (especially those you call “long term”, i.e., occurring outside the training context). This should be clearly acknowledged and the result interpretation modified.*

We agree with both the editor and Reviewer 1 and changed the text to reflect the fact that the differences we found between groups as regards demographic data can have also influenced the results and that both short- and long-term stressors can influence the cognitive bias results. This was included in the end of the Discussion section, where now it can be read “Because we did not randomly allocate dogs to the treatments (training methods), we cannot discard the possibility that there are significant differences between dog-owner pairs that lead some owners to choose an aversive-based school and others to choose a reward-based school. There were indeed differences among Groups in owner gender, in whether or not the household included children and in the information owners relied on for choosing the dog training school. There were also differences among Groups in dog age, breed and age of separation.” (lines 690-695) and “Thirdly, some caution is advised when interpreting cognitive bias results as unambiguous indicators of long-term welfare as they can be influenced both by short- and long-term stressors [37].” (lines 700-702)

3.2. *As well, in Figure 6, the data point for the Reward Group “N” position appears to be missing.*

We need to clarify that there was no data point missing in Figure 6. In this figure, the data points for the three groups overlap for the N and P positions due to the adjusted latency score we used, which set P as 0 and N as 1 for all subjects. In the current version of the manuscript, this information was added to the figure caption.

4. Cortisol measures
   4.1. *One of this issues with your averaging of cortisol levels by group is that the time of day training classes occurred and saliva samples were taken appears to vary widely among training schools/dogs. Please clarify if you think this had no effect, and why – for example, if the time of day for saliva collection between dogs in each group was about the same, then you could argue it had little effect.”*

We agree with the editor in that the time of day (which indeed varied among dogs and training schools) could have affected our cortisol levels. However, we corrected for this potential confounder when we used and analyzed the difference between PT and BL levels for each dog instead of the absolute PT and BL levels. This is also the reason why our Figure 5 reports the average difference between PT and BL levels instead of the absolute levels. Moreover, each individual dog always had saliva collected at the same time of the day for PT as well as BL.

4.2. *I do congratulate you on getting multiple samples for most of your dogs. However, your cortisol analysis is based on what might be considered a relatively small sample size (16 vs. 15 per group), and power is likely an issue as well.*

Although we agree that our cortisol analysis seems to be based on a relatively small sample size, during study design we performed sample size calculations. For salivary cortisol concentration these revealed that, for a power of 80%, the recommended sample size was n=5 per group, which is lower than our current sample size.

4.3. *Is there any relationship for the individual dogs sampled with the specific number of aversives used in training for those dogs? I suspect that the result reported on lines 543-544 uses the average number of aversives per school? Please comment.*

The results did indeed refer to the average number of aversive stimuli used in each school, but please refer to section 2.4 above.

4.4. *As well, Figure 5 is not compelling. Instead, the data on baseline vs. post-training CORT levels for the two groups would be better shown in the Figure.*

Please refer to section 4.1 above.

5. Terminology

*Reviewer 2 raises an important point about how you use the terms “positive punishment” and “negative reinforcement”. Please consider how best to address this, since its not likely that you have data to show that the techniques were actually reinforcing or punishing behaviour, based on their consequences. This will likely require re-wording throughout the manuscript and in Table S1b.*

We agree with the editor and Reviewer 2 and in the current version of the manuscript we changed the terminology to “intended positive punishment”, “intended negative reinforcement”, etc.

6. Inter-rater Reliability

*I commend you on the efforts you made to standardize behavioural coding across your three observers. However, it appears that, following training – which gave a high Cohen’s Kappa coefficient- the vast majority of videos were coded by the only observer (ACVC) who was not blind to the dogs’ condition/group. Following training, some percentage (~20% seems to be standard) of the videos coded by ACVC should have been coded by at least one other observer, and inter-rater reliability (e.g., intra-class correlation) for specific behaviours provided. If this was done, please provide these data. If this was not done, there is some concern that implicit bias from the “non-blind” coder could influence the data, and this must be addressed.*

Once again, we acknowledge we were not clear enough in the first version of the manuscript. Training involved coding of data from actual subjects, namely four videos for the ethogram for scan sampling and sixteen videos for the ethogram for continuous sampling. Hence, some percentage (although not 20%) of the actual data was coded by two observers. Namely, 1,5% of the data analyzed with the scan sampled ethogram was coded by ACVC and SP and 6% of the data analyzed with the continuous sampling ethogram was coded by ACVC and DF.

Reviewer #1 Comments

1. *The number of pairwise tests without corrections & the resulting inflated risk of Type II errors. Further, nonparametric tests such as pairwise comparisons & Friedman's don't allow for consideration of interactions. This is especially concerning as the authors discuss effects of training session on outcomes specific to groups (i.e. it appears there are indeed interactions, which makes discussion of main effects inappropriate). At a minimum, alpha corrections or multiple comparisons should be included. More elegant statistical approaches would benefit the manuscript. For example, 'panting' could have been considered using logistic regression with training group, training session, & group*session as independent variables.*

Please refer above to section 1 of Editor Comments.

2. *The (over)interpretation of the judgement bias paradigm used to infer long-term welfare effects. It would be nice to have the same or similar parameters compared both acutely and long-term (i.e. long term effects on cortisol & behavioral indicators), although I appreciate there are limitations with that approach. Cognitive bias paradigms are widely used as the authors state, but they are also widely over-interpreted. If one refers back to the original Mendl paper & the sources cited within, it becomes readily apparent that judgement biases can be influenced by *acute* stressors as well as long-term stress. That means it is not de facto evidence of long term affective states. The discussion does a nice job considering nuance in relation to other elements of the study, but presents no discussion of difficulties with interpretation of cognitive bias paradigms. This needs to be added to the discussion.*

Please refer above to section 3.1 of Editor Comments.

3. *Finally, lumping all schools that use *any* aversives together oversimplifies the approaches used by different training schools. While the reward based training schools used *no* aversives, there is a strong argument to be made that mainly positive punishment differs from truly balanced approaches that use as much or more positive reinforcement as aversives when training operant behaviors but also employ aversives when proofing already learned behaviors. The authors do discuss this nicely in lines 673-698, but this should be considered in the interpretation of the findings.*

Please refer above to section 2.3 of Editor Comments.

Reviewer #2 Comments

*1. I would argue that the terms positive reinforcement, negative reinforcement and positive punishment are used incorrectly throughout this paper. In Table S1b the authors define Positive Punishment as an “unpleasant stimulus applied to the dog” and Negative reinforcement as an “unpleasant stimulus that was applied and stopped”… However, in the field of behavior analysis, were these terms originated, reinforcement and punishment are not necessarily unpleasant or pleasant stimuli as viewed by an observer. For a stimulus to be considered punishment it must decrease a behavior and for it to be considered reinforcement it must increase a behavior. In this study data wasn’t collected on what happened to the behavior after these methods were implemented, thus we don’t know if punishment or reinforcement was used. I would suggest that the authors change these terms to something other than reinforcement and punishment so that it is not confusing to the reader*.

Please refer above to section 5 of Editor Comments.

2. *The authors stated in the methods that they collected the frequency of the methods and labeled the school aversive or reward based on the frequency that they observed. Those that used more aversive methods were labeled aversive and those that used more rewards were labeled rewarding. It would be helpful if we can see this frequency and a list of specific methods for the aversive and reward schools so that the reader has a better idea of how aversive and rewarding these schools were determined. I find this very important, as this is what the whole paper is based on.*

We believe that Table S1a in the present version of the manuscript contains the information requested by the reviewer. Additionally, in section *2.2.2. Classification of training methods* of the paper we describe in detail how the analysis of training methods was carried out.

*3. Line 140 “free of behavioral problems…” should clarify “free of certain behavioral problems” or you could use specific instead of certain.*

In the current version of the manuscript one can read “free of certain behavioral problems”. (line 166)

*4.*

*4.1. Line 116: the authors state that the first 15 minutes of three training sessions were used. Were these training sessions randomly picked?*

The three training sessions were chosen to occur early in training for each. As part of our inclusion criteria, and as reported in the paper, the first session needed to happen before the dog completed two months of training at the training school and the last session ought to occur within three months of the first one. Apart from that, which sessions were picked was mainly determined by the availability of the owner and the experimenters, which were collecting data at several schools at the same time.

*4.2. If so how many training sessions did the dog have during this study that you could have chosen from?*

Because the frequency of training sessions differed between schools (and even between dog owners within the same school), we decided to choose the time in training rather than the number of training sessions to define our criteria. Answering to the reviewer’s question in terms of time of training, the three-videotaped training sessions happened somewhere in-between three weeks and three months of training.
