# Supplementary material for: Does training method matter? Evidence for the negative impact of aversive-based methods on companion dog welfare
Source: PLoS One. 2020 Dec 16;15(12):e0225023. doi: 10.1371/journal.pone.0225023 (PMC7743949; doi:10.1371/journal.pone.0225023)
Supplement: Attachment — Submitted filename: Response to Reviewers.docx [file pone.0225023.s005.docx]

**RESPONSE TO EDITOR AND REVIEWERS**

We appreciate all the constructive criticism provided once again by the editor and the two anonymous reviewers. In our opinion, the manuscript as it currently stands has improved in both data analysis quality and strength of results. Generally, we included and tested confounders in our statistical models, re-analyzed the cognitive bias data with a modeling approach where we were able to test for an interaction between latency and bowl location, and reconsidered our claims on having assessed long-term welfare - in the current version of the manuscript cognitive bias is now treated as an indicator of welfare outside the training context. In what follows, we present detailed responses to all the comments.

Editor Comments

*1) Until you can convincingly show that the welfare differences you found are based on training school approach as the most compelling explanation, then your study will fall short of being able to advocate for the position that reward-based training generates the best welfare outcomes for dogs.*

As a consequence of not being able to randomly assign dogs to training schools, as you pointed out, we did end up with a dataset with some unbalanced distribution of possible confounders. We agree that it is important to show that the welfare differences are explained by the training school approach and are not artefacts of this distribution. We have addressed this by taking the confounders into account in the statistical analysis in the current version of the manuscript. We tested each confounder first, one at a time (i.e. 1 additional degree of freedom being used) in addition to our variables of interest, to verify if they were significantly related with the response variable and also if they substantially changed the independent variables’ estimates of the models (i.e. group and training session estimates). This way of testing confounders, in which they are tested one at a time, allows to maintain enough statistical power to verify their significance and influence in the models. If more than one confounder was found to be significant, then all the significant confounders were tested in the whole model. Non-significant confounders, variables of interest and interactions were removed from the final models. Only significant and trend models are presented in Appendix S5.

Prior to testing the confounders, we checked for multicollinearity issues among all confounders, as well as among confounders and dependent variables of interest. There were no collinearity issues between any of the factors, thus none of the confounders which we decided to test were removed from the analysis.

From the factors that differed among our groups, we decided to test Children (categorical, presence or not), Owner Gender (categorical, female or male) and Dog Age (numeric, months of age) as possible confounders, but not FCI Breed Group and Age of Separation from the Mother.

There is quite some literature suggesting that owner/handler gender and dog age can impact dog stress levels (e.g., Buttner et al, 2015; Henessy et al, 1998; Meyer and Forkman, 2014; Shöberl et al, 2016) and hence these two factors were tested as confounders. As regards Children, to our knowledge, there is no study directly studying or showing a relationship between the presence of children in the household and dog stress/welfare. However, a study by Meyer and Forkman (2014) showed that that children in the family was negatively associated with the owners' perception of the relationship with their dogs. As this can eventually result in a negative effect on dog welfare, we also decided to also include Children in the analysis. The reason not to include breed group is motivated by the incoherent relation between breed groups and behavior/temperament. It is known, as you referred, that breeds differ in behavior. However, when one tries to group breeds (either by kennel clubs’ conventional classification like the one we report in the present study, or through genetic relatedness) behavioural divergences of related dog breeds are found (e.g., Turcsán et al 2011). Mixed breeds make the issue even more complex. In order to include breed as a factor we would need to consider “mixed breed” as a breed group level, but that approach ignores the fact that this is a highly heterogenous group, where different individual dogs are related to completely different groups of pure breeds, and where in most cases we do not know which breeds they were crossed with (estimating dog breeds from appearance is not reliable, Voith et al 2009). Finally, we did consider testing Age of Separation from the Mother as a confounder, as it is documented that early separation can lead to stress and stress-associated behaviors in dogs (e..g, Tiira et al, 2012). However, we had a high amount of missing data for this factor (around 22% of the owners reported not knowing when the dog had been separated from the mother), which made it impossible for us to carry on with this test without the possibility of generating questionable results.

Results indicated that Presence of Children was a confounder affecting body turn and body shake in opposite directions. Dog Age was negatively associated with body turn and yawn. Owner Gender did not affect any of the studied variables. Although the significant confounders were kept in their respective models (as they should), we cannot draw any conclusions from an effect of a confounder. It is important to keep in mind that confounders correlate not only with the dependents variables but also with the INDEPENDENT VARIABLES of interest (once their parameter estimates were changed by more than 10% when confounders were added to the models). Thus, no conclusions can be drawn about why and how each of the significant confounders affected the dependent variables, as they are mere confounders, and also some of their results were not consistent among the behaviors. Similarly, we cannot conclude that Owner Gender, for example, is not relevant for explaining the behaviors studied. Our hypothesis did not include these possible confounders as variables of interest and the study was not designed to detect an effect of these, thus the possible confounders can and should only be used to improve the models mathematically.

To summarize, we appreciate this constructive criticism and wish to make it clear that the inclusion of the confounders improved some of the models explaining the dependent variables as a function of our treatments, while having only minor effects on the results. The effects of our variables of interest (Group and Training Session) remained robust and consistent, as you will find in the manuscript. There were minor adjustments on the p- and Z values. Following the comments by Reviewer 3, we re-analyzed the cognitive bias data with a modeling approach, and were able to test the same confounders to explain latency to reach the bowls. None of the confounders tested were significant in the cognitive bias task. Last, but not least, we added dog ID as a repeated measure in our behavioral (and cognitive bias) analysis, since we had each dog being subjected to 3 training sessions (and to 5 bowl locations). This probably helped to extract some of the variability caused by the unbalanced confounders and improved our models.

2) *I think that your statement from your reply regarding ‘de-briefing’ the instructors/owners at the training schools regarding the true goal of the study should be included in your section on Recruitment (2.2.1).*

This information has been included and this section now reads:

“Dog training schools within the metropolitan area of Porto, Portugal were searched on the internet. Eight schools were selected based on both their geographical proximity and on the listed training methods. Posteriorly, by telephone, head trainers were asked about their willingness to participate in a study to evaluate dog stress and welfare in the context of training and the methodological approach was thoroughly explained. Dog trainers, however, were not made aware that study results were going to be further compared among different training methods, to avoid any biases during training sessions. Of the eight contacted schools, seven agreed to participate. After study conclusion, a debriefing with the participating training schools was performed in order to communicate the results.”

3) *I believe you have adequately dealt with the part of comment #1 of Reviewer #3 regarding baseline cortisol measures (although, as the reviewer correctly points out, there are no ‘baseline’ measures prior to each training session- and this should be acknowledged in the paper).*

As we explain in point 1 in the response to Reviewer 3 comments below, there is a baseline physiological measure of stress outside training, through the saliva samples obtained by owners at home on non-training days. This is now mentioned in the last sentence in the 2^nd^ last paragraph of the Discussion: “Whereas we do not know the animals’ stress levels before the start of training, cortisol data shows no differences between training groups on non-training days.”

**Reviewer #1 comments**

*1) The authors did a nice job editing the text for language & overhauling the statistics section. I'm puzzled why a mixed model negative binomial regression was not used to control for the random effects of individual? This is a repeated measures design so partitioning that error is appropriate.*

This is correct, thank you for pointing that out. To make sure the repeated measures were necessary in the model, we compared the original models with new final ones with repeated measures (Clarke, 2007). Comparison results indicated that repeated measures were indeed appropriate and improved the original models. Therefore, we modeled the frequency of stress-related behaviors, behavioral states, and panting again as functions of the same treatments (Group and Training Session), their interaction and all possible confounders (as described in the methodology), WITH repeated measures. As you will notice, our new results are similar to the ones we had before and the new parameter estimates were updated in Appendix S5. Only significant and trend models are detailed in Appendix S5.

*2) Though they have made many changes to the manuscript, the cognitive bias issue has not been addressed in a substantive way. Because so much is made of assessing long-term welfare, I think it demands a more detailed examination of why they chose to substitute an entirely novel welfare indicator for Phase II without including any of the behavioral or physiological indicators used in Phase I. It bears discussing how a possibly transient negative affective state indicates long-term poor welfare.*

We rethought our claims on having assessed long-term welfare with the cognitive bias task, and the current version of the manuscprit advances a more less complex claim. Throughout the manuscript, we now refer to welfare within the training context (where we previously referred to short-term welfare effects) and outside the training context (where we previously referred to long-term welfare effects). Cognitive bias is now treated as an indicator of welfare outside the training context.

*3) This is a new comment, but I suggest including means + SEMs in the text of the results section. The graphs are difficult to see, and it seems odd to have to go the supplemental materials to view results used as dependent variables. A summary table with average occurrence by Group would be helpful.*

We have included means and SEMs for the stress-related behaviors as Figure 2 may actually be difficult to read, and also for the cortisol data that is not depicted in Figure 5 (namely, baseline and post-training levels). We considered including a table summarizing all the results, but this would result in redundancy of information. With the figures and the means and SEMs in the text for the data that may be difficult to read in the figures, we think we have adequatly dealt with this concern.

*4) It would be nice if the Discussion considered the uniformly low occurrence of stress indicators per training session... while statistically significant one wonders how biologically relevant some of these differences are (e.g. 3 versus 4 "move away")? Similarly I think the authors have captured nice data worthy of further consideration - e.g. what do they tell us about potentially robust indicators of acute stress?*

We agree that this is very interesting but in our opinion this study does not allow us to speculate about indicators of acute stress. The levels of stress-related behaviours are comparable to those found by Cooper et al (2014) in a similar study. However, the results of these two studies refer to a very specific context – that of dogs being trained for basic obedience. To establish robust indicators of acute stress would require a different kind of study, where dogs were subject to known stressors of a variety of types.

**Reviewer #3 comments**

Major concerns:

*1) Authors found that the stress-related behaviours changed across the three groups during the training. But we do not know what the dogs’ level of stress was before training. It is possible, therefore, that other factors may have led to stress-related behaviours. Consider for instance the living conditions of the dogs. Owners that choose specific training centres may have different approaches to the dogs when interacting with them in their home context. Moreover, several factors in training methods influence dogs’ performance and possibly their level of stress, like a tight schedule of reinforcement/punishment, the characteristics of the to-be-punished behaviour, and several other features. There is plenty of literature on the topic (I just mention one review chapter, but there are several others: Hineline, P. N., & Rosales-Ruiz, J. (2013). Behavior in relation to aversive events: Punishment and negative reinforcement. In G. J. Madden, W. V. Dube, T. D. Hackenberg, G. P. Hanley, & K. A. Lattal (Eds.), APA handbooks in psychology®. APA handbook of behavior analysis, Vol. 1. Methods and principles (p. 483–512)) which seems not only to be missing in the Introduction but also in the Authors’ hypothesis, as revealed by the absence of a baseline measurement for each participating dog.*

The study design with recruitment through training schools did not allow us to measure how the dogs behaved before they started attending training schools. We agree that other factors will affect stress levels, and we have approached this in different ways to minimize the risk of bias. Most importantly, we obtained baseline data on cortisol levels by instructing owners to take a cortisol sample at home at around the same time of day as training took place but on days where the dogs did not go to the training school (section 2.4.1). Baseline cortisol levels were similar between the three groups, indicating that the baseline levels of stress was not different between dogs being trained with different methods. Furthermore, we collected information about the dog and their living conditions through a questionnaire to the owners (section 2.5) so that we could see how these were distributed over treatment groups. Where a given situation was overrepresented in one treatment group, such as for example owner gender or the presence/absence of children, this was accounted for by including potential confounders in the statistical analysis (see the response to Editor comment 1 above). These considerations are now mentioned in the 2nd last paragraph of the Discussion. Whereas we agree that it would be interesting to consider the effect of ‘tightness’ of the schedule of reinforcement/punishment and stress-related behaviors in dog training, this was beyond the scope of the present study. By focusing on basic obedience training, we ensured that the behaviors under training were approximately the same across schools.

*2) The non-parametric test used for the Judgment Bias Paradigm does not allow to test the Group x Bowl Location interaction. This interaction would attest that the latency to approach the different locations of the bowl changed according to the training conditions. Therefore, it provides stronger evidence in support of your hypothesis. This interaction should be explored. On the topic, see Gygax (2014, doi: 10.1016/j.anbehav.2014.06.013).*

Thank you for bringing this up and for the reading suggestion. We used a generalized mixed model (GLMM) to evaluate raw latencies to reach the bowl as a function of our variables of interest (Group and Bowl Location), their interaction, and possible confounders (as described in the methodology), while accounting for the non-constant variability of our data. Raw latencies were used as suggested by Gygagx et al. (2014) and the individual variability among dogs was extracted from the model by adding Dog ID as a random effect, thus there was no need for adjusting the latencies with this approach. As you can see in the Results section, latency to reach the bowl was affected by both Group and Bowl Location, but not by their interaction, which was then removed from the final model. None of the tested confounders (Children, Owner Gender, and Dog Age) had a significant effect, possibly due to the effective blocking of dogs as a random effect, thus confounders were also removed from the final model. Model parameter estimates are presented in Appendix S5 submitted with the present manuscript.

*3) It is not clear to me whether the dogs had finished the training before doing the Judgment Bias task or they had just completed phase 1 of your experiment. If they had not completed the training, then it is misleading to claim that the Judgment Bias task assessed long-term welfare. In general, I think that discussing the results in terms of welfare within and outside the training context would be more appropriate because “short-“ and “long-term” are ambiguous concepts.*

The dogs had not necessarily finished the training (in fact most of them did not) and this is now made clearer in the text – now it reads “After finishing data collection for Phase 1, dogs participated in Phase 2, which consisted of a spatial cognitive bias task. The end of Phase 1 did not correspond to the conclusion of the training programs for the dogs, as this would result in different dogs being exposed to substantially different amounts of training before being assessed for cognitive bias. Instead, for standardization purposes, we ensured that 1) dogs had attended the training school for at least one month prior to Phase 2 and that 2) the cognitive bias task was conducted within one month of completing Phase 1.”

We have changed the terminology to describe the different aspects of welfare that were measured with the two methods. Throughout the manuscript, we now refer to welfare within the training context (where we previously referred to short-term welfare effects) and outside the training context (where we previously referred to long-term welfare effects).

Minor points:

*4) Could you please report the effect sizes of your results to facilitate future meta-analysis on this topic?*

We added a table on Supporting Information reporting the effect sizes (Appendix S4).

Finally, all the minor gramatical suggestions have been accepted.

References

1. Buttner AP, Thompson B, Strasser R, Santo J. Evidence for a synchronization of hormonal states between humans and dogs during competition. Physiol Behav. 2015; 147, 54‐62.
2. Hennessy MB, Williams MT, Miller D, Douglas CW, Voith VL. Influence of male and female petters on plasma cortisol and behaviour: can human interaction reduce the stress of dogs in a public animal shelter? Appl Anim Behav Sci. 1998; 61(1), 63-77.
3. Meyer I, Forkman B. Dog and owner characteristics affecting the dog–owner relationship. J Vet Behav. 2014; 9(4), 143-150
4. [Schöberl](https://www.ncbi.nlm.nih.gov/pubmed/?term=Sch%26%23x000f6%3Bberl%20I%5BAuthor%5D&cauthor=true&cauthor_uid=28178272) I, [Wedl](https://www.ncbi.nlm.nih.gov/pubmed/?term=Wedl%20M%5BAuthor%5D&cauthor=true&cauthor_uid=28178272) M, [Beetz](https://www.ncbi.nlm.nih.gov/pubmed/?term=Beetz%20A%5BAuthor%5D&cauthor=true&cauthor_uid=28178272) A, [Kotrschal](https://www.ncbi.nlm.nih.gov/pubmed/?term=Kotrschal%20K%5BAuthor%5D&cauthor=true&cauthor_uid=28178272) K. Psychobiological Factors Affecting Cortisol Variability in Human-Dog Dyads. [PLoS One](https://www.ncbi.nlm.nih.gov/pmc/articles/PMC5298213/). 2017; 12(2), e0170707.
5. Turcsán B, Kubinyi E, Miklósi A. Trainability and boldness traits differ between dog breed clusters based on conventional breed categories and genetic relatedness. Appl Anim Behav Sci. 2011; 132, 61–70.
6. Voith VL, Ingram E, Mitsouras K, Irizarry K. Comparison of adoption agency breed identification and DNA breed identification of dogs. J Appl Anim Welf Sci. 2009; 12(3), 253‐262.
7. Tiira K, Lohi H. Reliability and validity of a questionnaire survey in canine anxiety research. Appl. Anim. Behav. Sci. 2014; 155, 82–92.
8. Clarke, KA. A simple distribution-free test for nonnested model selection. Political Anal. 2007; 15(3), 347-363.
