# Supplementary material for: Does training method matter? Evidence for the negative impact of aversive-based methods on companion dog welfare
Source: PLoS One. 2020 Dec 16;15(12):e0225023. doi: 10.1371/journal.pone.0225023 (PMC7743949; doi:10.1371/journal.pone.0225023)
Supplement: Attachment — Submitted filename: Response to reviewers.docx [file pone.0225023.s006.docx]

**RESPONSE TO EDITOR AND REVIEWERS**

We appreciate all the constructive criticism provided once again by the editor and the reviewers. Our paper has become admittedly stronger with the review process. For the current version, we added information on the analysis of the confounders to the Statistical analyses section and also included a review of previous studies reporting an effect of these variables on dog behaviour and stress in the Discussion section. Moreover, the cognitive bias results are now discussed in more depth, with references to other studies that found similar results (i.e., differences also for training stimuli). Additionally, as requested, further information on how IRR was calculated is reported and some material that was in the appendices in the previous version of the paper was moved to the main text. All the remaining suggestions were also carefully considered. Detailed responses to all the comments are presented in what follows.

Editor:

***I. Confounds***

*I appreciate your comments in the response to reviewers letter regarding the fact that the confounders that appeared in your study- i.e., that the groups differed in variables other than those you are testing (training method), such as owner gender, dog breed, etc.- were not originally part of your hypotheses, and that you should not test specifically for them. I agree. It is an unfortunate fact that one difficulty in such “real world” research, in which we cannot randomly assign subjects to groups, is the presence of confounders which make our interpretation of our variables of interest tricky. If confounds are present (and they almost always are!), they have to be fully explicated, addressed statistically to the greatest extent possible, and then not forgotten when the data are interpreted. You have done each of these steps to a good degree, but I think a few more additions could improve the manuscript.*

*1) First, please consider whether in the Introduction you can introduce the notion that this is (what I call) a “real world” study, and you are not randomly assigning dogs/owners to training schools. Indicate that because of this, you will be evaluating for the presence of specific differences among the training groups that might influence to overall variables of interest. I believe this, in fact, is a strength of your paper, as you have taken considerable efforts to collect and analyze the demographic data for owners and dogs! However, while you mention the “Questionnaire” in the methods section and the data appear in the results, your efforts are not mentioned anywhere in the Introduction. It is fine to not have hypotheses about the questionnaire outcomes, of course, but I believe it would be good to highlight the care that you are taking in collecting such data, and your awareness that such factors could impact your group differences on the variables of interest.* ***This point was also raised in comment #1 by Reviewer #3.***

To clarify this, we have added the following two sentences to the second last paragraph of the introduction:

“We used a quasi-experimental approach in which dog-owner dyads were recruited to participate through the training school at which they were enrolled. As treatment could not be randomized, data on potential confounders was collected to be included in the analysis of treatment effects.”

*2) It would be useful to shorten what you write in response to the reviewers (under Editor Comments, 1), and put this in Section 2.7, to provide a fuller description of the choices you made regarding how you analyzed the “other” group differences (confounders) in relation to your variables of interest. This might also address the first part of comment #1 by Reviewer #3... i.e., if the training method still affects body turn, shake, yawn, and low state after controlling for potential significant confounders. (However, please see the entire comment and respond appropriately). It also might address the comment on “Statistical analysis” by Reviewer #4.*

We have added the following information to section 2.7, where the statistics analysis is described:

“To correct for the unbalanced distribution of potential confounders in the dataset, all known confounders for which sufficient data were available were considered in the analysis as follows. First, each confounder was tested, one at a time, in addition to the variables of interest, to verify if there was a significant relation between the confounder and the response variable, and if the confounder substantially changed the model estimate of the independent variables (i.e., group and training session estimates). This way of testing confounders, in which they are tested one at a time, allows to maintain enough statistical power to verify their significance and influence in the models. If more than one confounder was found to be significant, then all the significant confounders were tested in the whole model. Non-significant confounders, variables of interest and interactions were removed from the final models.”

*3) With respect to this reviewer’s comment on not including breed as a confounder, even though it differed significantly among groups, please address this more fully in the paper if you decide not to analyze it further. Currently, there is only one line in the Discussion (line 711-712) that dismisses the potential breed effect, which I- and likely many other readers- feel might be having an effect.*

We do not dispute that breed may have an effect on behaviour. Breed differences in behaviour are well established, but this applies to single breeds. Because of the heterogeneity of our sample, dogs were not classified according to single breeds but according to FCI breed groups. Moreover, 34% of the dogs were mixed breeds, mainly unknown. We have expanded on this question in the discussion where the relevant section now reads:

“Two of the potential confounders were not included in the analysis because of insufficient reliable data: breed (34% mixed breeds, mainly unknown) and age of separation from the mother (22% unknown). Breed differences in behavior are well established [43] but the classification of breeds into groups has not been found to systematically correlate with behavioral similarities [e.g., 45], and the large percentage of mixed breed dogs where the actual breeds were unknown further constrains a meaningful analysis of this factor in our sample. Literature shows that both early [e.g., 46] and late [e.g., 47, 48] separation from the mother (before and after 8 weeks-old, respectively) can be associated with stress-related behavioral problems in dogs.”

*4) For the results of the “Questionnaire”, I agree with Reviewer #3 that the interpretation of some of the differences in dog and owner demographics is difficult without looking in Appendix S3. However, instead of placing the appendix in the main text, I would recommend that for Sections 3.1.1 and 3.1.2, the direction for the significant results be placed in the main text by stating the medians/range (means/sd)– or proportions, whatever is most appropriate for the variable (vs. just the statistic and associated probability for each finding).*

The table is a frequency table and many of the variables have 5 or more categories, which makes it impossible to report the data as requested. We decided to follows Reviewer #3’ suggestion and condensed Appendix S3 to a table in the main text (now Table 4).

*5) Although you describe the possible effects of confounds as limitations in the Discussion, I think more exploration of these variables as alternate possible explanations for some of the findings- OR why they are NOT as strong explanations- is warranted. It is clear that your hypotheses are focussed on training method comparisons, and that should be the main focus of the Discussion. But engaging in some more discussion of how the ‘other’ group differences which appeared might also impact the behaviours during training, the cognitive bias findings, and the cortisol outcome (no difference) could be worthwhile and generate further research ideas.*

The potential confounders have been accounted for in the statistical analysis (except for breed and age of separation from the mother, where we had insufficient reliable data for a meaningful analysis), which means there is an effect of training method that is independent of the confounding effects of other variables. Whereas it would be misleading to discuss an effect of variables that the study was not designed to evaluate (**and for which we cannot thus infer any relation to our dependent variables**), we have added a review of previous studies reporting an effect of these variables on measures of dog behaviour and stress. The section of the discussion addressing the potential confounders now reads: “The study was not designed to evaluate the effect of these factors and they were therefore treated as potential confounders in the statistical analysis, in order to account for the possibility that they would affect our results. The effects of training method reported in the study are robust to these confounders. We tested for dog age, presence of children in the household and owner gender, factors which have been shown to potentially affect dog stress and welfare [e.g., 41-44]. The presence of children in the family has been found to be negatively associated with the owners' perception of the relationship with their dogs, in what is to our knowledge the only study addressing how this factor affects dog behavior [43]. Most research into the relationship between dog age and stress indicators has been conducted in senior dogs and consistently shows higher baseline cortisol and higher cortisol responses to stressful stimuli in aged dogs [45, 46]; however, our study did not include any senior dog. [Schöberl](https://www.ncbi.nlm.nih.gov/pubmed/?term=Sch%26%23x000f6%3Bberl%20I%5BAuthor%5D&cauthor=true&cauthor_uid=28178272) et al (2017) [44] found cortisol to decrease with increasing age of the dog in adult dogs, whereas Henessy et al (1998) [42] found that the juveniles and adults had higher plasma cortisol levels than puppies**.** Two of the potential confounders were not included in the analysis because of insufficient reliable data: breed (34% mixed breeds, mainly unknown) and age of separation from the mother (22% unknown). Breed differences in behavior are well established [43] but the classification of breeds into groups has not been found to systematically correlate with behavioral similarities [e.g., 47], and the large percentage of mixed breed dogs where the actual breeds were unknown further constrains a meaningful analysis of this factor in our sample. Literature shows that both early [e.g., 48] and late [e.g., 49, 50] separation from the mother (before and after 8 weeks-old, respectively) can be associated with stress-related behavioral problems in dogs.”

*Also, the comment by Reviewer #4 that owner-dog interactions during a training session might reflect daily non-training interactions should be integrated into the Discussion as well.*

See response to Reviewer #4 below.

**II. Cognitive Bias Outcomes**

*The new analysis in line with Gygax’s (2014) recommendations is sound. However, it is not clear to me that your interpretation of its meaning is! As I understand the concept of cognitive bias, it is specifically the difference in behaviour towards the “ambiguous” stimulus (M, in this case) which is interpreted as either a more optimistic or a more pessimistic bias. In your data, the Group Aversive dogs responded to ALL the food bowl locations more slowly, and the latency to bowl M did not change for this group relative to their response to the other bowls. This is interesting indeed and might indicate that in the Group Aversive dogs, there is more behavioural inhibition or the like. However, is it accurate to call this pessimism? Can you please either address this issue in the manuscript and support your interpretation with some citations that also interpret latency to perform “all” (vs. just ambiguous) tasks as a pessimistic bias, or update how this cognitive bias finding is interpreted in the manuscript?*

*Currently, given the new cognitive bias findings, I believe there is NOT much solid support for any welfare effect outside the context of training, as the cortisol shows no differences in “non-training day” measures. So, supporting your current interpretation of the cognitive bias outcome is critical for your argument to stand.  If you cannot sufficiently bolster the cognitive bias interpretation as above, it might be necessary to pull back from claims about “poorer welfare” for dogs exposed to aversive training classes, and instead focus on your strong effects, which are the group differences emerging from the behaviour coded during training sessions, and the post-training cortisol levels.*

Although affect is hypothesised to exert a greater influence on decision-making under ambiguity (test stimuli: NN, M, NP) than under certainty (training stimuli: N, P), some studies in cognitive bias have found differences for both test and training stimuli (e.g., Deakin, 2018; Horváth et al, 2016; Zidar el at, 2018, see Neville et al 2020 for a review). This type of result has also been interpreted as evidence for differences in the valence of the affective states. The fact that differences can emerge for both training and test stimuli has been proposed to result from the fact that choice in the cognitive bias task depends on two different components of the decision-making process: perceived probability and perceived valuation of rewards (and punishments). An individual may be less likely to make a less “risky” or more “pessimistic” response if they consider the reward to be less probable (or punisher more probable) and/or if they consider the reward to be less valuable (or the punisher more aversive) (Iigaya et al, 2016; Neville et al, 2020). In the current study, Group Aversive displayed higher latencies for all the stimuli (training and test). Therefore, the most likely explanation for our findings is that dogs from Group Aversive considered the food reward less probable (as indicated by the higher latencies to the test cues) and also showed a higher valuation of reward loss relative to win (as indicated by the higher latencies to the P and N bowls) (Iigaya et al, 2016). Because similar findings have been interpreted as indicative of more “pessimistic” responses, we are therefore confident that our results reflect a real difference in the affective states of the dogs from Group Aversive vs. the dogs from Groups Reward and Group Mixed. These ideas appear now in the Discussion section:

“When considering welfare outside the training context, we found that, in the cognitive bias task, dogs from Group Aversive displayed higher latencies for all the stimuli than dogs from Group Reward, with no differences being found between Groups Aversive and Mixed nor between Groups Reward and Mixed. Although affect is hypothesized to exert a greater influence on decision-making under ambiguity (i.e., for the test stimuli: NN, M, NP) than under certainty (i.e., for training stimuli: N, P), other studies in cognitive bias have also found differences for both test and training stimuli [e.g., 32-35, see 35 for a review]. This type of result, with differences found for (at least one of) the training stimuli has also been interpreted as evidence for differences in the valence of the affective states. The fact that differences can emerge for both training and test stimuli has been proposed to result from the fact that choice in the cognitive bias task depends on two different components of the decision-making process: perceived probability and perceived valuation of rewards (and punishments). An individual may be less likely to make a less ‘risky’ or more ‘pessimistic’ response if they consider the reward to be less probable (or punisher more probable) and/or if they consider the reward to be less valuable (or the punisher more aversive) [35,36]. In summary, affective states may influence the responses to both the training and the test stimuli in the cognitive bias task, although different components of the decision-making process may be playing a role. Therefore, the most likely explanation for the present findings is that dogs from Group Aversive considered the food reward less probable (as indicated by the higher latencies to the test stimuli) and also showed a higher valuation of reward loss relative to win (as indicated by the higher latencies to the training stimuli) [36]. Overall, these results indicate that dogs from Group Aversive were in a less positive affective state than dogs from Group Reward.”

Regarding the baseline cortisol levels vs. the cognitive bias findings, please refer to our response to Reviewer 3 comment 1b) below.

**III. Inter-rater Reliability**

*There is still a lack of clarity on how strong inter-rater reliability (IRR) for the behavioural measures actually is, as pointed out by Reviewer #4.  It is critically important for you to be able to convince readers that there is acceptable/high inter-rater reliability for these behaviours, as the behaviour effects are some of your strongest. Currently, as you report it, there were 3 observers coding videos, only 2 of which were blind to the group assignment of the dogs. The first observer, who was NOT blind to condition, was responsible for coding the vast majority of the videos. This, in and of itself, is not necessarily a problem, IF you can demonstrate convincingly that there is high IRR for each behaviour coded among the observers. This requires reporting: 1) the total number of videos watched/coded and the percentage of videos coded by each observer, and 2) for each behaviour, a value for IRR (whatever statistic best suits your situation) - which can be presented as an appendix. Without this additional information, we are unable to ascertain the extent to which it is possible that unconscious bias in coding by the non-blinded observer might have influenced the outcome. So, please augment this section. If it requires additional coding by observers, this is worthy investment in time and effort.*

Our method of video analysis and of IRR calculation followed that performed by Cooper et al (2014). As in their study, “Each observer received training to become familiar with the ethogram developed (…) to allow assessment of inter-observer reliability. Inter-observer reliability was tested by allocating four videos to different observers at an early stage of analysis. Consistency in scoring was assessed by calculating the correlation coefficient r (…) Where r>0.8, it was assumed there was good agreement between observers’ scores and they were reliably following the sampling method. Where there was poor agreement (r<0.8), observers received further training to address inconsistencies.”

In our study, IRR was calculated for the entire ethogram and not for each behavioural category. We changed the text accordingly to make this information clear and also to report all the additional requested data. Now it reads:

“The second and fourth authors were trained to become familiar with the ethograms and inter-observer reliability was assessed for each ethogram by having the corresponding pair of observers watch and code sets of four videos at an early stage of analysis. Cohen’s Kappa coefficient was calculated for each pair of videos using The Observer XT. After analyzing each set of four videos, if there was poor agreement for any video (r<0.80), the observers received further training. Values of r>0.80 were assumed to indicate strong agreement, and once this level was attained for the four videos of the set, the observers began coding videos independently [9]. A total of 265 videos were coded. For the ethogram for continuous sampling, the analysis of 16 videos was needed before a value of r>0.80 was achieved, whereas for the ethogram for scan sampling, r>0.80 was achieved after analysis of 4 videos. Afterwards, for each ethogram, the remaining videos were distributed randomly between observers, while ensuring that each observer coded a similar percentage of videos from each experimental group. The first author coded 76% of the videos with the ethogram for stress-related behaviors and 64% with the ethogram for overall behavioral state and panting.”

**IV. Effect sizes:**

*Inclusion of effect sizes is excellent, as pointed out by reviewers. However, they are lost in the Appendix. Please include them in the main text, with each result reported. The magnitude of the effect sizes for the behaviours is a strength!*

We have incorporated the effect size into the main text – see also our response to Reviewer 3 comment 3 below.

**V. Appendix vs. In-text:**

*Both reviewers recommend moving some of the information in the Appendices to the main text. It is my preference that the ethograms/behavioural definitions appear in the main text in a table, not in an appendix. However, for the other appendices, I believe it is “author’s choice”.*

The following tables were moved from the Appendices to the main text:

Table S1a (Appendix S1) is now Table 1.

Appendix S2 is now Table 2 and Table 3.

Appendix S3 is now condensed in Table 4.

Reviewer #3:

*a) I appreciate the fact that you have considered several potential confounders. Since you have found that the three groups differ along several demographic factors, you should report which group is different in the main text - not only in the SI. It is otherwise hard to interpret the results from line 472 to 475.*

Please check our answer to concern 4) from the Editor.

*Also, and more importantly, you should check if the training method still affects Body turn, Body shake, Yawn and Low State after controlling for the potential significant confounders.*

It is not clear to us what the reviewer’s concern is. However, all the results reported in the current and previous versions of this manuscript refer to the effect of training method after controlling for the confounders.

*b) I am still not convinced that the measurement of cortisol levels during - and not before - Phase 1 can be considered as a reliable baseline. In fact, in Phase 2, you demonstrated that the training method had affected dogs' welfare outside of its immediate context. Why should cortisol levels not be affected in the same way?*

We appreciate this comment and apologize if through the revision process we have contributed to a misunderstanding of the purpose of the baseline cortisol measure. We never intended the cortisol level on non-training days to be used as an indicator of overall stress level. The purpose of determining saliva cortisol in each dog on non-training days was to be able to determine the acute cortisol response to training. We do not intend to make any claim in the manuscript of having a baseline measure of general stress level, but if you (reviewer or editor) think that this is the case, we would appreciate if you would point out where and we will revise accordingly.

In addition, it is quite consensual that salivary cortisol is not a reliable measure of long-term stress. Importantly, the effects of chronic stress on physiological data and other responses (such as behavior) can be contradictory [e.g., Pawluski J, Jego P, Henry S, Bruchet A, Palme R, Coste C, et al. (2017) Low plasma cortisol and fecal cortisol metabolite measures as indicators of compromised welfare in domestic horses (Equus caballus). PLoS ONE 12(9): e0182257. <https://doi.org/10.1371/journal.pone.0182257>].

*c) Also, I still think it is important for a potential reader to understand that several factors involved in training methods might have affected your outcome variables. These factors may also have affected your results. You should at least mention this in the Introduction.*

We have added a reflection on other factors which may influence dogs in the Introduction, where the 2^nd^ paragraph now reads as follows, referring to Hineline & Rosales-Ruiz (2013) as previously suggested by this reviewer:

“Dog training most often involves the use of operant conditioning principles, and dog training methods can be classified according to the principles they implement: aversive-based methods use mainly positive punishment and negative reinforcement and reward-based methods rely on positive reinforcement and negative punishment [3]. Within a given training method, several factors may influence how dogs react, such as the characteristics of the behavior under training and the timing of reinforcement/punishment [4]. However, the use of aversive-based training methods *per se* is surrounded by a heated debate, as studies have linked them to compromised dog welfare [5-10].”

*2) Thanks for your reply, I believe that you have properly addressed this point.*

*3) Thank you for clarifying this point. Because some dogs had finished the training while others had not, I wonder if the three groups differed in the number of training sessions they had attended before Phase 2.*

This information is in section 3.3, where it reads:

“Prior to the cognitive bias task, dogs from Group Aversive, Mixed, and Reward attended (M±SEM) 6.29±0.47, 7.14±0.65 and 6.07±0.36 training classes, respectively, with no significant differences observed among groups [H(2)=2.7, p=0.258].”

*4) Thank you for adding the effect-sizes.*

Reviewer #4:

*1) I have read the paper and past reviews with interest and commend the authors on their work. It is indeed difficult to disentangle the many factors potentially affecting dogs’ welfare. While previous studies regarding relationships of owners’ training style and dog welfare have been mostly correlational, this manuscript has several strengths, as designation of training methods, as well as the welfare indicators were done based on objective measures and not owner report. The authors used a multimodal approach – behavioural indicators of acute stress, cortisol measures as well as the judgement bias test. I also appreciate that it takes a lot of effort to recruit and test a sample size of 92 dogs.*

We appreciate this recognition of the strengths of the study – thank you!

*2) The revised statistics appear to be well-founded, and the authors appropriately acknowledge the limitations of their study. Clearly there are many influencing factors that can affect a dog’s daily welfare. Nonetheless, it would not be unreasonable to assume that owners’ interactions during the training session are indicative of their interactions during everyday life, and this could potentially explain the differences in the cognitive bias tests. This concern (according to reviewer 3 of the last round of reviews) is actually something I would view as an advantage, with the results likely not only having implications for the time the dog spends in dog school, but potentially the everyday interactions with their owners. Probably this should be discussed, as different reviewers independently brought this up.*

We appreciate this comment and agree that one would expect that the way owners interact with their dogs during training sessions reflect at least to some extent how they interact during everyday life. Indeed, it is usually part of the training schools’ objective that owners continue to implement what they learn during lessons in all kinds of interactions with their dog. As this reviewer observes in the previous paragraph, the present study is centred on objective measures collected by the research team, and we did not collect data on dog-owner interactions outside the training school. Therefore, we would rather not speculate on the relation between dog-human interactions inside and outside the school as regards our sample. We recognize that the issue is important and have added a reflection in the last paragraph of the discussion, which now ends “This applies not only to training in a formal school setting but whenever owners use reinforcement or punishment in their interactions with the dog.”

*3) Effect sizes (Cohen’s d) reported in the appendix were large, as many were >1. I think it would be worth pointing out in the main text that there were large effect sizes, which is even more informative than the p values.*

We have incorporated effect size as Cohen’s d in the results section, so that each comparison now reads something like “Group A was higher/lower than Group B (Z=4.6, p<0.001, d=1.02).

*Abstract:
One of the study’s strengths, in my opinion, is that training method was objectively measured. Since not everybody reads the whole paper, I would recommend to include this information in the abstract such as was stated in the Introduction “By performing an objective assessment of training methods (through the direct observation of training sessions) and by using objective measures of welfare (behavioral and physiological data to assess effects during training, and a cognitive bias task to assess effects outside training)”*

We appreciate this comment, but the word limit for the abstract does not allow more information to be added. The data collection method is already described in detail in the abstract.

*Line 29: I don’t think the authors can claim to have investigated the “entire range of aversive-based techniques (beyond shock-collars)”. Rather, it is relevant that the observed intended positive punishments were presumably less aversive than shock collars, and still clear differences between the groups were found. So I would rather frame it such that previous studies used very highly aversive stimuli such as shock collars which may not be relevant to most dogs’ everyday lives, whereas the observed techniques were.*

This part of the text has been removed as the abstract was condensed.

*Line 104: “we addressed the question of whether aversive-based methods actually compromise the well-being of companion dogs” - Perhaps it would be beneficial to state this in a more neutral way such as “assessed the effects of reward-based and aversive-based methods on welfare of companion dogs”. Although welfare is unlikely to be influenced by time in the training school alone, it is likely to reflect on the everyday interaction of the dogs and owners*

This has been reworded to “assessed the effects of reward-based and aversive-based methods on companion dog welfare”

*Line 125: term “posteriorly” – I believe you mean “Prior to inclusion in the study”, rather than after?*

This has been reworded so that the description of recruitment now reads “The head trainers were invited by telephone to participate in the study. They were informed that the aim was to evaluate dog stress and welfare in the context of training and the methodological approach was thoroughly explained. To avoid bias during recorded training sessions, the trainers were not made aware that study results were going to be further compared among different training methods.”

*Line 147: include a reference for the statement “In order to be coherent with the standard for classification of operant conditioning procedures as reinforcement or punishment (which is based not on the procedure itself but on its effect on behavior).*

We have included a reference to Skinner (1953) – Science and Human Behavior.

*Line 155: I feel it is important how the schools were designated as aversive or reward based, so personally I would prefer to have this information in the main manuscript, rather than the appendix.*

In order to include more detail on how schools were designated, we have incorporated what was previously Table S1 into the main text, as Table 1.

*Line 327: As above, I would prefer to know the details of behaviour codings to assess welfare from the paper, rather than the appendix.*

In order to provide details of how behaviour was coding, we have incorporated what was previously Tables S2a and S2b into the main text, as Tables 2 and 3.

*Line 337: it is not totally clear to me on the basis of how many videos reliability was assessed at the end, and what percentage of videos was coded by each of the coders.*

Please check the answer to the Editor’s comment above.

*Statistical analysis*

*Line 397: Why were confounders tested one at a time and not simply included in the full model? (I realise it might possibly be due to power/ sample size if too many variables are included in the model?).*

The reviewer is correct. Now this is directly explained in the text in section 2.7, where it reads:

“This way of testing confounders, in which they are tested one at a time, allows to maintain enough statistical power to verify their significance and influence in the models. If more than one confounder was found to be significant, then all the significant confounders were tested in the whole model. Non-significant confounders, variables of interest and interactions were removed from the final models.”

*While I wouldn’t insist on it, in my opinion including breed in the model might be worthwhile. The authors commented that they found doing this not useful given that mixed breeds are not a homogenous group. There are, however some potentially relevant systematic difference also between mixed breeds and purebreds: Turcsán, B., Miklósi, Á., & Kubinyi, E. (2017). Owner perceived differences between mixed-breed and purebred dogs. PloS One, 12(2), e0172720.
Riemer, S. (2019). Not a one-way road – severity, progression and prevention of firework fears in dogs. Plos One, 14(9), e0218150.*

We appreciate this comment and do not dispute that breed may affect behaviour. However, a meaningful analysis of a breed effect on behaviour requires a categorization of dogs into breeds for which there is consistent information on behaviour. Our sample is too heterogeneous for a classification into single breeds (many of which are represented by only one dog), and so we used the FCI breed categories. Classification of breeds into groups has not been found to systematically correlate with behavioral similarities, and the large percentage of mixed breed dogs where the actual breeds were unknown further constrains a meaningful analysis of this factor in our sample, as we are explaining in the discussion.

*Line 426: Effect sizes could be reported in the results, rather than the appendix*

This has been changed – see also our response to Reviewer 3 comment 3 above.

*Line 538: maybe “require” instead of “take”? (English suggestions)*

This has been changed as suggested.

*Line 619: maybe “possibly reflects” instead of “is possibly a reflex of”*

This has been changed as suggested.

*Line 658: also one year since the “treatment” is a long time for this to still have an effect*

It is not clear to us if the reviewer is suggesting a change to the text here.

*Discussion: perhaps it could be discussed that the cognitive bias test indicates welfare differences between the three groups, but this was not reflected in baseline cortisol measures*

As explained in detail in our response to Reviewer 3 comment 1b) above, baseline cortisol data in this study was collected to estimate the acute cortisol response to training and not to estimate general stress level. Additionally, check the cited reference for salivary cortisol not being considered as a reliable measure of long-term stress.

*Line 714: However, recent studies show that adoption >8 weeks is also associated with a higher incidence of behaviour problems than adoption at 8 weeks
Jokinen, O., Appleby, D., Sandbacka-Saxén, S., Appleby, T., & Valros, A. (2017). Homing age influences the prevalence of aggressive and avoidance-related behaviour in adult dogs. Applied Animal Behaviour Science.*

*Puurunen, J., Hakanen, E., Salonen, M. K., Mikkola, S., Sulkama, S., Araujo, C., & Lohi, H. (2020). Inadequate socialisation, inactivity, and urban living environment are associated with social fearfulness in pet dogs. Scientific reports, 10(1), 1-10.*

Thank you for these useful references. We have reworded this sentence to “Literature shows that both early [e.g., 48] and late [e.g., 49, 50] separation from the mother (before and after 8 weeks-old, respectively) can be associated with stress-related behavioral problems in dogs.”

*Appendix 1:*

*I would suggest to write “presumably unpleasant”/ “presumably pleasant” stimulus, rather than having “unpleasant” or “pleasant” in parentheses.*

This has been changed in what is now Table 1 in the manuscript.

*I was wondering how often petting the dog was observed compared to feeding? (as being petted might not necessarily be perceived as pleasant in a training context, even if it is meant as reward by the human)*

This is a really interesting comment and we totally agree. Although we haven’t formally analysed this data, School A exclusively used petting as R+ as opposed to the remaining schools for which food was the R+ of election (petting was only occasional). This most likely explains why this school had the highest levels of stress behaviors among schools using aversive-based methods (data not presented do). We now address this issue in the Discussion. See next comment.

*I would appreciate a full list of all behaviours included in the definitions of “pleasant” and “unpleasant”, and perhaps their frequencies. Perhaps the current Appendix 1 could go into the main text, and the frequencies of different types of pleasant and unpleasant stimuli in the Appendix.*

Part of Appendix 1 is already placed as a table in the main text. We have also added to the table the full list of stimuli used in training (we believe this was what the reviewer meant, not behaviours). Additionally, we added the following in the Discussion:

“Moreover, our results suggest that the proportion of aversive stimuli used in training plays a greater role on dogs’ stress levels than the specific training tools used. As an example, one school from Group Mixed used pinch and e-collars, whereas other school from Group Aversive only used choke collars during training. Although the tools used by the former school may be perceived as more aversive, the frequency of stress behaviors was higher in dogs being trained at the latter school. The type of (intended) positive reinforcers also appears to be relevant. All schools except the aforementioned school from Group Aversive used primarily food treats as rewards, whereas the latter only used petting. Although this was not the school using the highest proportion of aversive stimuli, it was the school whose dogs showed the highest frequency of stress behaviors (data not shown). Previous research has shown that petting is a less effective reward than food in training [40]. Having a highly valuable reward might thus be important in reducing stress when aversive stimuli are used in training. The goal of the present study was to test the overall effect on dog welfare of aversive- and reward-based methods as they are used in the real world, but it may be interesting for future studies to focus on disentangling the effects of the different types of stimuli used in training (as has been done with e-collars) [e.g., 9, 25].”

In order to prevent the individual schools from being identifiable through their specific training techniques, we decided not to mention to which specific schools we are referring to in this paragraph.

*Appendix 2:*

*I think in the definition for move away it should read “dog takes” not “dog gives”*

This has been changed in the table, which is now Table 2 in the main text.

*The visible lines seem to be slightly mixed up for vocalisations.*

We are sorry but we cannot figure out what the reviewer is referring to. We would be thankful if you could point it out so that we can make the corrections accordingly.

*Paw lift: “for a brief or a more prolonged time” is very unspecific.*

This was changed to “One fore limb only is lifted, usually in a slow movement, and either immediately returned to rest on the ground or remaining lifted for a brief period.”, as the new definition is more specific and still reflects what was agreed between the two observers that coded the video with this ethogram.

*Fig 3 and 4 differ in that there are lines in Fig 3 at the x-axis and between labels but not 4 in the same position.*

Once again, we are sorry but we cannot figure out what the reviewer is referring to, as in both figures the lines separate the results of the three groups. We would be thankful if you could point it out so that we can make the corrections accordingly

*General: I found some double empty spaces in the text, which can be found with the search and replace function*.

Thank you for pointing this out; the extra empty spaces have been deleted.
